# Environmental and Genetic Factors Involved in Plant Protection-Associated Secondary Metabolite Biosynthesis Pathways

**DOI:** 10.3389/fpls.2022.877304

**Published:** 2022-04-08

**Authors:** Xiaori Zhan, Zhehao Chen, Rong Chen, Chenjia Shen

**Affiliations:** ^1^College of Life and Environmental Sciences, Hangzhou Normal University, Hangzhou, China; ^2^Zhejiang Provincial Key Laboratory for Genetic Improvement and Quality Control of Medicinal Plants, Hangzhou Normal University, Hangzhou, China; ^3^School of Public Health, Hangzhou Normal University, Hangzhou, China

**Keywords:** abiotic stress, biotic stress, plant specialized metabolites, plant protection, transcription factor

## Abstract

Plant specialized metabolites (PSMs) play essential roles in the adaptation to harsh environments and function in plant defense responses. PSMs act as key components of defense-related signaling pathways and trigger the extensive expression of defense-related genes. In addition, PSMs serve as antioxidants, participating in the scavenging of rapidly rising reactive oxygen species, and as chelators, participating in the chelation of toxins under stress conditions. PSMs include nitrogen-containing chemical compounds, terpenoids/isoprenoids, and phenolics. Each category of secondary metabolites has a specific biosynthetic pathway, including precursors, intermediates, and end products. The basic biosynthetic pathways of representative PSMs are summarized, providing potential target enzymes of stress-mediated regulation and responses. Multiple metabolic pathways share the same origin, and the common enzymes are frequently to be the targets of metabolic regulation. Most biosynthetic pathways are controlled by different environmental and genetic factors. Here, we summarized the effects of environmental factors, including abiotic and biotic stresses, on PSM biosynthesis in various plants. We also discuss the positive and negative transcription factors involved in various PSM biosynthetic pathways. The potential target genes of the stress-related transcription factors were also summarized. We further found that the downstream targets of these Transcription factors (TFs) are frequently enriched in the synthesis pathway of precursors, suggesting an effective role of precursors in enhancing of terminal products. The present review provides valuable insights regarding screening targets and regulators involved in PSM-mediated plant protection in non-model plants.

## Introduction

The ability of plants to synthesize an extremely wide arsenal of diverse metabolites makes them preeminent chemists (Fernie et al., 2004). Traditionally, plant metabolites are classified into two groups: primary and secondary (Patra et al., 2013). Primary metabolites are ubiquitous in all plants and play crucial housekeeping roles in plant growth and development (Fabregas and Fernie, 2021). Secondary metabolites, also called plant specialized metabolites (PSMs), are involved in various physiological and biochemical processes, such as defense and adaptation to adverse environments (Chapman et al., 2019; Sic Zlabur et al., 2021). With the development of detection technology, more PSMs have been identified and characterized in plants.

On the basis of their core structures, PSMs form three major categories: nitrogen-containing chemical compounds, terpenoids/isoprenoids, and phenolics (Marone et al., 2022). Nitrogen-containing compounds, consisting of cyanogenic glycosides, alkaloids, and glucosinolates, have been widely identified in natural plant products and synthetic compounds (Aharoni and Galili, 2011). Terpenoids/isoprenoids can be divided into five subgroups: monoterpenes, sesquiterpenes, diterpenes, triterpenes, and tetraterpenes, on the basis of the number of isoprene structural units (Bohlmann et al., 1998). Phenolics, containing at least one aromatic ring and one hydroxyl group, can be divided into four functional classes: phenolic acids, flavonoids, tannins, and stilbenes (Aharoni and Galili, 2011; Rasouli et al., 2016). Most of PSMs are produced by individual metabolic pathways and unequally accumulate in different tissues and organs. The structural complexity and uneven distribution ensure different biological functions of PSMs under changing environmental conditions (Desmet et al., 2021).

The roles of PSMs in human health and their potential as pharmaceutical drugs have been studied extensively (Hamilton, 2004; Tungmunnithum et al., 2018). Medicinal plants produce valuable PSM-derived drugs, such as, taxol from *Taxus media*, quinine from *Cinchona officinalis*, withanolide from *Physalis angulate*, and artemisinin from *Artemisia annua*, are widely applied in the treatment of a variety of serious diseases (Ravishankara et al., 2001; Zhan et al., 2018; Shi et al., 2021; Wani et al., 2021; Yu et al., 2021). In plants, PSMs are essential for several physiological processes, such as plant protection, pollinator attraction, signal transduction, and seed germination, which are required for their survival in harsh environments (Chae et al., 2014; Liu et al., 2021d; Singh et al., 2021; Wari et al., 2021).

Plant specialized metabolites contribute to plant protection against different types of biotic and abiotic stresses, similar to the adaptive immune system in animals (Castro-Moretti et al., 2020; Desmet et al., 2021). To adapt to stress conditions, plants gear their metabolism toward the biosynthesis of PSMs, which, although energy-costly, is beneficial for their survival (Abdala-Roberts et al., 2016). PSMs serve in various parts of a complete plant defense system, acting as message molecules and/or antioxidants (Kumar and Pandey, 2013). Some PSMs act as key components of complex signaling pathways and trigger both the extensive expression of defense-related genes and the accumulation of other metabolites (Maag et al., 2015). Other PSMs serve as antioxidants, participating in the scavenging of rapidly rising reactive oxygen species (ROS) and in the chelation of heavy-metal ions under stress conditions (Pacifico et al., 2021). They not only act as powerful antioxidants, they may also be toxic to herbivores, microbial pathogens, and competing plant species (Glas et al., 2012). Overall, some of the credit for a plant’s capability to tolerate or adapt to a changing environment goes to PSMs. The purpose of this review is to summarize the basic biosynthetic pathways of protection-related PSMs on a limited scale. Furthermore, the environmental and genetic factors involved in the biosynthesis of PSMs are also briefly summarized.

## Basic Biosynthetic Pathway of Plant Specialized Metabolites

There are more than 400,000 vascular plants with up to one million metabolites on the Earth (Fang et al., 2019). Although there are many PSMs, their chemical structures are not random (Fernie et al., 2004). The vast majority of PSMs are variations on a core derived from several typical backbones having structural modifications, such as glycosylation, acylation, methylation, hydroxylation, and prenylation (Wang et al., 2019c). On the basis of the representative structures, PSMs can be grouped into major classes, such as nitrogen-containing compounds, terpenoids, and phenolics (D’Auria and Gershenzon, 2005). Here, we summarize the basic biosynthetic pathways of representative PSMs and attempt to better understand the potential targets of stress-mediated regulation.

### Key Enzymes Involved in the Biosynthesis of Alkaloids

Alkaloids originally consisted of a large class of heterocyclic nitrogen-containing organic compounds (Zhang et al., 2021b). The nitrogen atom in the heterocyclic ring generally originates from an amino acid. On the basis of their amino acid precursors and chemical structures, alkaloids are classified into five subgroups: terpenoid indole alkaloids (TIAs), benzylisoquinoline alkaloids (BIAs), tropine alkaloids, purine alkaloids, and pyrrolizidine alkaloids (Bhambhani et al., 2021). From precursors to final products, a series of biochemical modification reactions occur during the different alkaloidal conversion steps, ensuring diverse arrays of chemical structures and biological activities (Lichman, 2021; Zhang et al., 2021c). The present review uses TIA and BIA as examples to investigate the complexity of alkaloid biosynthesis.

Terpenoid indole alkaloids are a class of PSMs found in various non-model medicinal plants, such as *Catharanthus roseus*, *Rauvolfia serpentina*, *Ophiorrhiza pumila*, and *Vinca minor* (Luijendijk et al., 1996; Schlager and Drager, 2016; Shi et al., 2020a; Zhan et al., 2020; Yang et al., 2021a; Vrabec et al., 2022). *Catharanthus roseus* is frequently used as the model plant to reveal a complete TIA biosynthetic pathway (O’Connor and Maresh, 2006). Strictosidine, a key skeleton unique to TIAs, is synthesized by strictosidine synthase with tryptamine, an indole ring donor derived from decarboxylated tryptophan, and secologanin, a terpenoid donor from the methylerythritol 4-phosphate (MEP) pathway (Moreno et al., 1993; Rai et al., 2013; Kumar et al., 2015). A series of key enzymes, including geraniol synthase, geraniol 10-hydroxylase, 10-hydroxygeraniol oxidoreductase, iridoid synthase, iridoid oxidase, 7-deoxyloganetic acid glucosyltransferase, 7-deoxyloganic acid, loganic acid-O-methyltransferase, and secologanin synthase, are involved in TIA skeleton biosynthesis (Geu-Flores et al., 2012; Simkin et al., 2013; Shen et al., 2017; Sandholu et al., 2020; Jeena et al., 2021). Then, the intermediate strictosidine is modified by different enzymes to produce species-specific TIAs (Qu et al., 2018; Williams et al., 2019). As detection technology progresses, more novel TIAs and TIA-related enzymes are being identified in different plant species.

Benzylisoquinoline alkaloids are also members of a structurally diverse class of PSMs that mainly exist in the *Ranunculales* order (Ziegler and Facchini, 2008). The biosynthesis of BIAs starts with dopamine and 4-hydroxyphenylacetaldehyde, a tyrosine derivative, to produce a fundamental precursor trihydroxylated alkaloid (*S*)-norcoclaurine by norcoclaurine synthase (Sheng and Himo, 2019). *O*-methylation, *N*-methylation, and hydroxylation successively occur on 4-hydroxyphenylacetaldehyde to synthetize (*S*)-3′-hydroxy-*N*-methylcoclaurine (Liu et al., 2021c). The conversion of (*S*)-3′-hydroxy-*N*-methylcoclaurine to (*S*)-reticuline, a branch-point product, in the production of morphine, tetrahydropalmatine, sanguinarine, and noscapine, is conducted by 3′-hydroxy-*N*-methylcoclaurine 4′-hydroxylase (He et al., 2018). Finally, cytochrome P450 superfamily proteins are responsible for several modification reactions, such as hydroxylation, isomerization, and coupling, on the BIA backbone that produce species-specific BIAs (Hori et al., 2018; Menendez-Perdomo and Facchini, 2018).

### Key Enzymes Involved in the Biosynthesis of Glucosinolates

As a class of wound-induced PSMs, glucosinolates highly accumulate in the Brassicaceae family of plants (Sanchez-Pujante et al., 2017). Most of glucosinolates can be grouped into three major subgroups, aliphatic, indole-, and aromatic glucosinolates, on the basis of their amino acid features (Ishida et al., 2014). The complete biosynthetic pathway of glucosinolates in *Brassica* genus consists of three steps: side-chain elongation, core structure formation, and side-chain secondary modifications (Sotelo et al., 2016).

During side-chain elongation, aliphatic and aromatic amino acids are utilized to produce 2-oxo acids by branched-chain amino acid aminotransferase family enzymes. Then, 2-oxo acid and acetyl-CoA are condensed by methylthioalkylmalate synthase (Kochevenko et al., 2012). Side-chain elongation ends with an isomerization process and an oxidative decarboxylation by isopropylmalate isomerase and isopropylmalate dehydrogenase, respectively (Sanchez-Pujante et al., 2017). To produce the core structure of glucosinolates, the conversion of side-chain-elongated amino acids to aldoximes is catalyzed by cytochrome P450 mono-oxygenases, such as CYP79 and CYP83, to produce *S*-alkyl-thiohydroximate and thiohydroximate (Robin et al., 2016). Then, thiohydroximate is catalyzed to form the glucosinolate core structure by two key enzymes, uridine diphosphate glycosyltransferase 74 and sulfotransferases (Sonderby et al., 2010; Robin et al., 2016). Finally, side-chain modifications, such as oxidation, hydroxylation, methoxylation, alkenylation, and benzoylation, are required for the formation of the terminal glucosinolate products (Halkier and Gershenzon, 2006; Nguyen et al., 2020).

### Key Enzymes Involved in the Biosynthesis of Terpenoids

Terpenoids are a structurally diverse group of PSMs in which each member has a core isoprene unit. The central core of the terpenoids is synthesized by one-unit dimethylallyl diphosphate (DMAPP) and three-units isopentenyl diphosphates (IPP) (Yu et al., 2017). Both DMAPP and IPP originate from the MEP pathway, occurring in the plastids, and from the mevalonate pathway, occurring in the cytoplasm, endoplasmic reticulum, and peroxisomes (Mahmoud et al., 2021).

Here, we take model plant *Arabidopsis* as an example. Isopentenyl phosphate kinase, common to most plants, catalyzes the conversion of isopentenyl monophosphate and dimethylallyl monophosphate to IPP and DMAPP (Henry et al., 2015). In plants, there is a classic upstream pathway that forms prenyl diphosphates having varied chain lengths, such as geranyl diphosphate (GPP), having 10 isoprene units, farnesyl diphosphate (FPP), having 15 isoprene units, and geranylgeranyl diphosphate (GGPP), having 20 isoprene units (Tholl, 2015; Jia and Chen, 2016). Next, terpene synthases participate in the conversion of FPP, GGPP, and GPP into mono-/sesqui-terpenes (Pichersky and Raguso, 2018; Zhou and Pichersky, 2020). Two units of FPP and one unit of GGPP can be condensed by squalene synthase to produce squalene and by phytoene synthase to produce phytoene, which are the precursors of sterols and carotenoids, respectively (Christianson, 2017). Thousands of different terpenoids having the same core skeleton are produced by various modifications, such as hydroxylation, dehydrogenation, reduction, glycosylation, methylation, and acylation (Liu et al., 2022a).

### Key Enzymes Involved in the Biosynthesis of Phenolics

#### Phenolic Acids

Phenolic acids are important active ingredients in numerous medicinal plants (Chen et al., 2021b). Bioactivities, biosynthesis and biotechnological production of phenolic acids have been well revealed in *Salvia miltiorrhiza* (Shi et al., 2019). On the basis of the number aromatic ring structures, phenolic acids can be classified into different groups (Choi et al., 2021). Taking *S. miltiorrhiza* as an example, most phenolic acids are synthesized through the phenylpropanoid and tyrosine metabolic pathways (Wang et al., 2015). In the phenylpropanoid pathway, phenylalanine is treated as a substrate to produce cinnamic acid by phenylalanine ammonia-lyase (PAL) (Reyes Jara et al., 2022). Then, cinnamic acid is catalyzed to *p*-coumaroyl-CoA by two enzymes, cinnamic acid 4-hydroxylase (C4H), and 4-coumarate: CoA ligase (Huang et al., 2008). In the tyrosine pathway, tyrosine aminotransferase and hydroxyphenylpyruvate reductase are involved in the conversion of tyrosine to 3,4-dihydroxyphenyllactic acid (Rizi et al., 2021).

Subsequently, rosmarinic acid, an important precursor for downstream species-specific phenolic acids, is synthetized by rosmarinic acid synthase and cytochrome P450-dependent monooxygenase CYP98A14 (Deng et al., 2020b; Chen et al., 2021b). Over-expression of rosmarinic acid synthase and CYP98A14 resulted in higher content of phenolic acids in *S. miltiorrhiza* hairy roots (Fu et al., 2020).

#### Flavonoids

Flavonoids are a class of water-soluble pigments stored in the cell vacuoles (Dong and Lin, 2021). In plants, more than 9,000 flavonoids have been identified and classified into different groups on the basis of the number of hydroxyl/methyl groups on their heterocyclic or benzene ring (Noda et al., 2017). As phenolics, flavonoids also originated from the phenylpropanoid pathway (Wang et al., 2018b). Specific flavonoid biosynthesis starts with the conversion of *p*-coumaroyl-CoA, together with malonyl-CoA and acetyl-CoA, to naringenin chalcone by chalcone synthase, which is the first rate-limiting enzyme in the flavonoid biosynthetic pathway (Zhang et al., 2017). Naringenin chalcone, a basic skeleton for the downstream pathway, is converted to naringenin by the catalysis of chalcone isomerase, or it is converted to naringenin chalcone 2’-glucoside by the catalysis of chalcone 2’-glucosyltransferase (Miyahara et al., 2018).

Chalcone is a central intermediate product in different branch pathways, such as the flavanone biosynthesis, flavone biosynthesis, isoflavone biosynthesis, and flavonol biosynthesis (Liu et al., 2021b). In the cytoplasm, chalcone isomerase participates in the cyclization of chalcones to produce flavanones, opening a route to the heterocyclic C-ring-containing flavonoids (Nabavi et al., 2020). In addition, naringenin is the precursor for eriodictyol biosynthesis by flavanone 3′-hydroxylase catalysis, and for pentahydroxyflavanone biosynthesis by flavanone 3′,5′-hydroxylase catalysis (Grotewold, 2006). Flavone biosynthesis is another branch of the flavonoid biosynthetic pathway. Flavone synthase catalyzes the conversion of flavanones to flavones, such as apigenin, dihydroxyflavone, luteolin, and tricetin (Zuk et al., 2019). Flavanones can also be converted to apigenin *C*-glycosides and luteolin *C*-glycosides by flavanone-2-hydroxylase (Lam et al., 2019).

Multiple metabolic pathways have the same origin, and the common enzymes are frequently to be the targets of metabolic regulation. The MEP pathway provides common precursors for the TIA biosynthesis and terpenoid biosynthesis pathways. The phenylalanine pathway provided common precursors for the phenolic acid biosynthesis and flavonoid biosynthesis pathways. Manipulation of these common enzymes affects multiple metabolic pathways to response to environmental stresses.

## Effects of Environmental Factors on Plant Specialized Metabolites Biosynthesis

The synthesis and tissue-specific accumulation of PSMs are strictly controlled in spatio-temporal mode and affected by various biotic and abiotic factors (Yang et al., 2012). Environmental stresses influence the formation and accumulation of PSMs in plants (Gupta and Dutta, 2011).

### Effects of Environmental Factors on Alkaloid Biosynthesis

Over a hundred TIAs, such as bisindole alkaloids, have been detected in the medicinal plant *C. roseus* (Shukla et al., 2006). *Catharanthus roseus* seedlings under drought- and salinity-stress conditions exhibit a greatly higher alkaloid content compared with under control conditions (Hassan et al., 2021; Yahyazadeh et al., 2021). Furthermore, the impact of drought and salt stresses on the biosynthesis and accumulation of alkaloids, such as dihydrocoptisine, has also been revealed in *Chelidonium majus* (Yahyazadeh et al., 2018). Cadmium chloride elicitation increases the yields of reserpine and ajmalicine, two important MIAs, in the endangered medicinal plant *Rauvolfia serpentina* (Zafar et al., 2020).

Under herbivore attack, the biosynthesis of physostigmine, an approved antiherbivore alkaloid, rapidly increases in the damaged area (Rivero et al., 2021). Various stresses elevate the content of a mixture of toxic pyrrolizidine alkaloids in *Echium plantagineum* plants, protecting them from insect and livestock herbivory (Skoneczny et al., 2019). Aphid predation induces the biosynthesis of quinolizidine alkaloids, a type of toxic secondary metabolites produced in lupin species (Frick et al., 2019). As a type of PSM, both of biotic and abiotic stresses up-regulate the content of alkaloids, suggesting their important roles of in resistance to environmental stress.

### Effects of Environmental Factors on Glucosinolate Biosynthesis

Glucosinolates are important precursors to various active ingredients in the Brassicaceae family of plants (Moreno et al., 2006). In pak choi (*Brassica rapa*), strong light, high-temperature, and drought increase the accumulation of glucosinolates (Park et al., 2021; Rao et al., 2021). *Brassica oleracea* has a powerful tolerance to chilling and freezing, and the low temperature-induced content of glucosinolates is hypothesized to be involved in the protective mechanism that enables this tolerance (Ljubej et al., 2021). In addition to low temperature, other postharvest stresses, such as wounding, also induce the biosynthesis of glucosinolates in *B. oleracea* (Villarreal-Garcia et al., 2016). In *Broccoli* sprouts, both of UV-A and UV-B light doses affect the tailored glucosinolate and phenolic profiles, suggesting an important role for light stress in glucosinolate biosynthesis (Moreira-Rodriguez et al., 2017).

Biotic stresses also can influence the glucosinolate composition in plants. The aphid-induced expression of *CYP79B2*, *CYP79B3*, and *PAD33* leads to the accumulation of indolyl glucosinolates (Mewis et al., 2012). A gain-of-function *Arabidopsis* mutant, *cml42*, with a higher aliphatic glucosinolate content than the wild type, shows a strong resistance to herbivory (Vadassery et al., 2012).

### Effects of Environmental Factors on Terpenoid Biosynthesis

Terpenoids play frequent roles in plant protection in the form of phytohormones, particularly as diterpene gibberellins, triterpene brassinosteroids (BRs), and sesquiterpene abscisic acid (ABA) (Patra et al., 2013; Chen et al., 2021a; Liu et al., 2021a). In lettuce, long-term high temperature expose facilitates the accumulation of gibberellin to accelerate bolting (Liu et al., 2020). Under drought-stress conditions, significant accumulations of ABA occur in wheat guard cells (Wang et al., 2021). In maize sprouts, NaCl stress greatly increases the content of carotenoid, which is a typical tetraterpenoid with an intense antioxidant capacity, by up-regulating the expression of several carotenoid biosynthetic pathway genes (He et al., 2021). In winter wheat, cold treatments elevate the endogenous BR content, indicating a role of triterpene BR in improving the cold tolerance of winter cereals (Janeczko et al., 2019).

Biotic stresses can also influence the terpenoid contents of plants. In various Iranian cultivars of basil, water-deficit stress enhances the accumulations of linalool, germacrene D, and γ-cadinene, three important aromatic terpenes with verified cytotoxic activities (Khakdan et al., 2021). The ability to synthesize specialized antimicrobial avenacins, belonging to the triterpenoids, is likely to have allowed oats (*Avena* spp.) to combat various diseases (Qi et al., 2004). Terpenoids are rich in *Euphorbia peplus* latex and function as defensive chemical substances against insect herbivores and various agricultural phytopathogenic fungi (Hua et al., 2017).

A large number of works showed that terpenoids are involved in the resistance to environmental stress in the form of phytohormones. Phytohormones, as signal molecules, transmit environmental signals to plant cells.

### Effects of Environmental Factors on Phenolic Acid Biosynthesis

The biosynthesis of phenolic compounds is significantly affected by various abiotic stress conditions (Sharma et al., 2019). In many inbred maize lines, long-term drought treatments cause significant reductions in various phenolic acids, such as protocatechuic, caffeic, and sinapic (Kravic et al., 2021). In Chinese cabbage, salt stress leads to great decreases in phenolic compounds, such as sinapic acid, salicylic acid, and ferulic acid (Linic et al., 2021). Under alkaline conditions, rice enhances phenolic acid secretions in the root epidermis and stele, which effectively increases ion uptake and alleviates the Fe-deficiency responses (Li et al., 2021b). In *Achillea pachycephala*, drought stress dramatically increases the contents of phenolic acids, such as chlorogenic and caffeic (Gharibi et al., 2019).

Rhizobacteria-mediated systemic resistance helps protect plants from pathogens and insects (Singh et al., 2002). Phenolic acid-induced systemic resistance provides bio-protection to plants under pathogenic stress conditions (Nicholson and Hammerschmidt, 2003). In rice, the correlation between phenolics and seedling protection from *Rhizobium solani* has been revealed. An High Performance Liquid Chromatography analysis showed that the biosynthesis of phenolic acids is more enhanced in Rhizobium-infected seedlings compared with uninfected controls (Mishra et al., 2006). In the orchid *D. officinale*, a *Dendrobium* viroid infection increases the total phenolic acid content, which may play an important role in the activation of pathogen defense responses (Li et al., 2022).

Phenolic acid has complex biological functions. Some stresses inhibit phenolic acid synthesis, and some other stresses promote phenolic acid contents, suggesting that phenolic acids may play both positive and negative roles in the process of resisting environmental stress.

### Effects of Environmental Factors on Flavonoid Biosynthesis

Flavonoids, common polyphenols, are antioxidants required in plant stress resistance (Laoue et al., 2022). Plants with high flavonoid contents have potential cellular antioxidant capacities under environmental stress conditions (Hidayat and Wulandari, 2021). The over-accumulation of several flavonoids, such as kaempferol, quercetin, and cyanidin, has been well documented in model plants (Nakabayashi et al., 2014). In Chinese liquorice, the contents of some ortho-dehydroxylated B-ring flavonoids, effective scavengers of ROS, increase under UV-B exposure (Zhang et al., 2018). In rice, salt and heat stresses enhance flavonoid accumulation, which is crucial for stress tolerance (Jan et al., 2021). In some plant species, abiotic stresses play negative roles in flavonoid accumulation. For example, accumulated Na^2+^ in *Apocynum venetum* leaves reduces the flavonoid concentration and decreases salt tolerance under salt-stress conditions (Xu et al., 2021).

The protective roles of hesperidin and hesperetin, the major flavonoids in citrus fruit, against invading microbes and toxins have been well investigated (Iranshahi et al., 2015). Several flavonol glycosides, such as quercetin and kaempferol glycosides, increase under short-wavelength radiation, which enhances plant defenses against various herbivorous insects (Rechner et al., 2017).

Anthocyanins, another subgroup of flavonoids, are frequently induced in plants by biotic and abiotic stresses (Li et al., 2021a). Various environmental factors play distinct roles in anthocyanin biosynthesis and tissue-specific accumulation in plants (Li et al., 2012; An et al., 2020). In apple, drought, low temperature, UV-B, and light exposure significantly up-regulate the accumulation of anthocyanins in fruit, and high temperature and increased nitrogen fertilizer significantly down-regulate the accumulation of anthocyanins in fruit (Gao et al., 2021).

## Effects of Genetic Factors on the Biosynthesis of Plant Specialized Metabolites

Accumulations of PSMs under stressful environment conditions is controlled by an intricate network containing a large number of Transcription factors (TFs). Many key enzyme-encoding genes involved in PSM biosynthesis are the downstream targets of different TFs (Patra et al., 2013).

### Transcription Factors Involved in the Biosynthesis of Alkaloids

Previous studies have identified several TFs that control specific steps and branches of the TIA and BIA biosynthetic pathways. In *C. roseus*, an ORCA3 TF regulates the expression of TIA biosynthetic pathway-related genes, such as *GEISSOSCHIZINE SYNTHASE*, *STRICTOSIDINE SYNTHASE*, and *DEACETYLVINDOLINE ACETYLTRANSFERASE* (Khataee et al., 2020). The interaction of MYC2 and GBFs governs TIA biosynthesis by modulating the TIA pathway genes in *C. roseus* (Sui et al., 2018). WRKY1 is a positive regulator of the TIA biosynthetic pathway (Suttipanta et al., 2011). A MAP kinase cascade modulates the TIA biosynthetic pathway by activating its downstream target *AP2/ERF* TF genes (Paul et al., 2017). In addition, the zinc-finger TF ZCT1 acts as a transcriptional repressor in the TIA biosynthetic pathway (Mortensen et al., 2019). In *Ophiorrhiza pumila*, OpWRKY2 and OpWRKY3 were identified as two positive regulators in the biosynthesis of camptothecin (Wang et al., 2019a; Hao et al., 2021).

In lotus (*Nelumbo nucifera*), WRKY40a and WRKY40b participate in the BIA biosynthetic pathway by regulating the *TYDC*, *NCS*, *CYP80G*, and *7OMT* genes (Meelaph et al., 2018; Li et al., 2019a). In narrow-leafed lupin, the TF RAP2-7 is involved in the regulation of the quinolizidine alkaloid biosynthetic pathway (Czepiel et al., 2021). Two jasmonate-responsive TFs, ERF189 and ERF199, are involved in the biosynthesis of nicotine, the predominant alkaloid in tobacco leaves (Kato et al., 2014; Kajikawa et al., 2017). Under high temperature-stress conditions, MYC2 enhances the nicotine content by regulating the expression of the *PMT1* gene, which encodes a putrescine *N*-methyl transferase involved in the key step of the pyridine alkaloid pathway (Yang et al., 2016). In *Coptis japonica*, isoquinoline alkaloid biosynthesis is controlled by CjbHLH1 homologs (Yamada et al., 2011).

### Transcription Factors Involved in the Biosynthesis of Glucosinolates

*Arabidopsis* is a model plant used to reveal the transcriptional regulation of glucosinolate biosynthesis (Hirai et al., 2004). An analysis of the R2R3-MYB family in *Arabidopsis* showed that MYB34, MYB51, and MYB122 control the biosynthesis of indolic glucosinolates, whereas MYB28, MYB29, and MYB76 control the biosynthesis of aliphatic glucosinolates (Frerigmann and Gigolashvili, 2014; Baskar and Park, 2015). Another two *Arabidopsis* TFs, FRS7 and FRS12, are transcriptional repressors in the glucosinolate biosynthetic pathway (Fernandez-Calvo et al., 2020). In addition, a well-identified central circadian clock regulator, CCA1, participates in the host resistance of plants to the caterpillar *Trichoplusia ni* by enhancing basal indole glucosinolate biosynthesis (Lei et al., 2019). A bHLH TF, IAA-LEUCINE RESISTANT3, modulates the accumulation of glucosinolates under iron deficiency conditions and during pathogen infection (Samira et al., 2018). A proteomic analysis identified a jasmonate-responsive MYC2 TF that has opposite effects on the indolic and aliphatic glucosinolate pathways (Guo et al., 2012).

Short-term temperature treatments can enhance the accumulation of glucosinolates in *B. rapa*. A co-expression analysis identified a MYB family member, MYB51, that regulates the biosynthesis of glucosinolates after a short-term high temperature treatment (Rao et al., 2021). In addition, MYB28.3, MYB29.1, and MYB122.2, which are highly responsive to various abiotic and biotic stresses, are positive regulators of aliphatic glucosinolate biosynthesis in *B. rapa* (Baskar and Park, 2015; Seo et al., 2017).

### Transcription Factors Involved in the Biosynthesis of Terpenoids

A number of TFs are involved in the terpenoid biosynthetic pathway (Patra et al., 2013). Artemisinin is an important sesquiterpene lactone in sweet wormwood, and several artemisinin biosynthesis-related TFs have been identified (Efferth, 2017). In sweet wormwood, two JA responsive TFs, ERF1 and ERF2, affect artemisinin biosynthesis by regulating the expression of *AMORPHA-4,11-DIENE SYNTHASE* and *CYP SEQUITERPENE OXIDASE* genes (Yu et al., 2012). AaWRKY1 controls the expression of *3-HYDROXY 3-METHYLGLUTARYL-COA REDUCTASE* and *ARTEMISINIC ALDEHYDE*Δ*11(13) REDUCTASE*, which are key genes in the artemisinin biosynthetic pathway (Jiang et al., 2016).

Several stress-related TFs are involved in the biosynthesis of terpenoids. In cotton (*Gossypium arboretum*), GaWRKY1 regulates the conversion of sesquiterpenes to gossypol, which plays a role in responses to fungal infection (Xu et al., 2004). Terpenoids are enriched in the latex products from the rubber tree (*Hevea brasiliensis*). HbWRKY1 and HbEREBP1 are positive and negative regulators, respectively, of latex biosynthesis induced by wounding (Chen et al., 2012; Wang et al., 2013). Clade Iva bHLH TFs in the JA-signaling pathway participate in the regulation of bioactive terpenoid biosynthesis (Mertens et al., 2016b). In *Medicago truncatula*, two bHLH TFs, TSAR1 and TSAR2, affect triterpene saponin biosynthesis by regulating the expression of *HMGR1*, which encodes the rate-limiting enzyme for triterpene biosynthesis, under stress conditions (Mertens et al., 2016a). In roses, the over-expression of the *PAP1* TF gene significantly activates the terpenoid biosynthetic pathway to enhance the production of terpenoid scent compounds (Zvi et al., 2012). The JA-responsive TF WRKY24 promotes the biosynthesis of saponin by increasing the expression of terpenoid biosynthetic pathway genes in *Conyza blini* (Sun et al., 2018). In *Taxus media*, a phloem-specific MYB3 affects the transcriptional regulation of paclitaxel biosynthesis, a classic diterpenoid compound, by activating the expression of *TBT*, *DBTNBT*, and *TS* genes (Yu et al., 2020).

### Transcription Factors Involved in the Biosynthesis of Phenolic Acids

Several TFs act as regulators of the phenolic acid pathway in the Chinese medicinal plant *S. miltiorrhiza* (Sun et al., 2019a). A large number of TFs, including two ERF family members (SmERF115 and SmERF1L1), three MYB family members (SmMYB2a, SmMYB2b, and SmMYB52), four bHLH family members (SmbHLH3, SmbHLH37, SmbHLH51, and SmbHLH148), one ZIP family member (SmZIP1), and two GRAS family members (SmGRAS1 and SmGRAS2), are involved in the regulation of the phenolic acid biosynthetic pathway (Zhou et al., 2016; Du et al., 2018; Li et al., 2019b; Deng et al., 2020a; Zhang et al., 2020). Furthermore, the corresponding downstream targets of the above TFs also have been identified in *S. miltiorrhiza*. SmMYC2a/b binds to the E-boxes in the promoter regions of *SmHCT6* and *SmCYP98A14*, which are key genes involved in the synthesis of 4-coumaroyl-3′,4′-dihydroxyphenyllactic acid and rosmarinic acid, respectively (Zhou et al., 2016). SmGRAS1, together with SmGRSA2, binds to the GARE box in the promoter region of *SmKSL1*, which catalyzes the biosynthesis of tanshinones from GGPP (Li et al., 2019b). SmbZIP1, an ABA-responsive TF, binds to the G-Box-like1 motif in the promoter region of *SmC4H1*, which is involved in the biosynthesis of phenolic acid precursors (Deng et al., 2020a). SmbHLH148 and SmMYB1 regulate phenolic acid biosynthesis by activating the expression of downstream genes, such as *PAL1*, *C4H1*, *TAT*, *HPPR*, *RAS*, and *CYP98A14* (Xing et al., 2018; Zhou et al., 2021b). SmMYB52 simultaneously affects the production of phenolic acids by binding to the MBE elements in promoter regions of *SmTAT1*, *Sm4CL9*, *SmC4H1*, and *SmHPPR1* (Yang et al., 2021b). Furthermore, SmbHLH3 acts a repressor in the biosynthesis of phenolic acids in *S. miltiorrhiza* hairy roots by reducing the expression of *DXS3*, *DXR*, *HMGR1*, *KSL1*, *CPS1* and *CYP76AH1* (Zhang et al., 2020). In *S. miltiorrhiza* hairy roots, tanshinone and salvianolic acid biosynthesis are controlled by SmMYB09 (Hao et al., 2020). Interestingly, over-expression of *Arabidopsis* MYC2 simultaneous promotes the biosynthesis of tanshinone and phenolic acid in *S. miltiorrhiza* hairy roots (Shi et al., 2020b). Additionally, SmJRB1 was identified as a positive regulator in regulation of phenolic acid biosynthesis (Zhou et al., 2021a).

### Transcription Factors Involved in the Biosynthesis of Flavonoids

The complete flavonoid biosynthetic pathway, consisting of three major branches, has been well-studied in plants. Recently, TFs from different families, such as MYB, bHLH, and WRKY, have also been characterized (Lloyd et al., 2017; Yan et al., 2021).

In *M. truncatula*, MtMYB134 activates flavonol biosynthesis by binding the promoters of *MtFLS1*, *MtFLS2*, and *MtCHS2* (Naik et al., 2021). In apple, MdNAC52 regulates the biosynthesis of anthocyanin and proanthocyanidin by activating the promoters of *MdMYB9* and *MdMYB11* genes (Sun et al., 2019b). In buckwheat, MYBF1 regulates the flavonol biosynthetic pathway by up-regulating the *DFR* and *LDOX* genes (Matsui et al., 2018). In *Fagopyrum tataricum*, light-induced FtMYB116 promotes the accumulation of rutin by binding directly to the promoter region of *F3’H* (Zhang et al., 2019). In pear (*Pyrus pyrifolia*), PpMYB17 positively controls the flavonoid biosynthetic pathway by activating the promoters of *PpCHS*, *PpCHI*, *PpF3H*, *PpFLS*, and *PpUFGT* (Premathilake et al., 2020). Another pear (*Pyrus* × *bretschneideri*) TF, PbWRKY75 affects flavonoid biosynthesis by regulating the expression of *PbDFR*, *PbUFGT*, and *PbMYB10b* (Cong et al., 2021). In *Populus tomentosa*, PtMYB6 promotes anthocyanin and proanthocyanidin biosynthesis by interacting physically with KNAT7 (Wang et al., 2019b). In potato (*Solanum tuberosum*), StWRKY13 promotes anthocyanin biosynthesis in tubers by activating the promoters of *StCHS*, *StF3H*, *StDFR*, and *StANS* (Zhang et al., 2021a). The over-expression of MdWRKY11 in apple calli revealed its novel function in promoting the accumulation of flavonoids and anthocyanin by binding to the promoter of *MdHY5* (Wang et al., 2018a; Liu et al., 2019).

Furthermore, many negative regulators of the flavonoid biosynthetic pathway have been identified in different plant species. AtMYB4 and its close homologs AtMYB7 and AtMYB32 inhibit flavonoid accumulation by down-regulating the expression of *ADT6*, which catalyzes the key step that supplies phenylalanine (Wang et al., 2020). The over-expression of *MYB15L* in red-fleshed apple calli represses anthocyanin accumulation and cold tolerance (Xu et al., 2018). Homodimers of MdMYB16 inhibit anthocyanin synthesis through their C-terminal EARs, which are weakened by interactions with the TF MdbHLH33 (Xu et al., 2017). The over-expression of *Arabidopsis* MYB60 in lettuce plants significantly reduces the production and accumulation of anthocyanin pigments by inhibiting the expression of *DIHYDROFLAVONOL-4-REDUCTASE* gene (Park et al., 2008). The loss of *MYB2-1* expression causes the purple color in cabbage leaves, suggesting that it encodes a potential negative regulator of the flavonoid biosynthetic pathway (Song et al., 2018). In *Ginkgo biloba*, a negative regulator, GbMYBF2, affects flavonoid biosynthesis by down-regulating several key genes, such as *GbPAL*, *GbANS*, *GbFLS*, and *GbCHS2* (Xu et al., 2014). A *Brassica napus* WRKY TF, BnWRKY41-1, acts as a repressor of anthocyanin biosynthesis (Duan et al., 2018). In *S. miltiorrhiza*, SmbHLH60 was identified as a negative regulator in anthocyanin biosynthesis mainly *via SmDRF* gene (Liu et al., 2022b).

**FIGURE 1:**
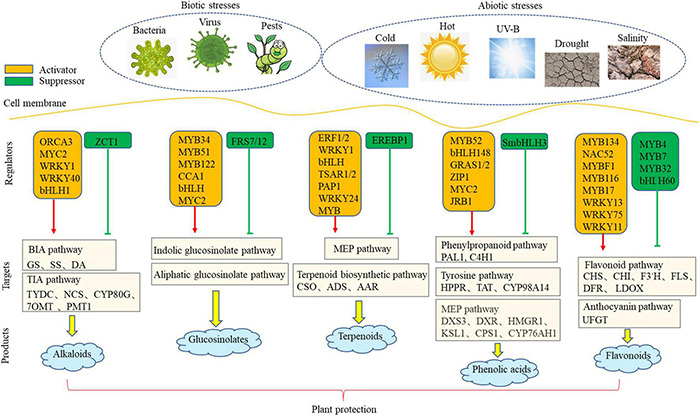
The network of transcriptional regulation of plant under biotic and abiotic stresses. Red arrow indicated activation pathways and green arrow indicated inhibition pathways.

Environmental signals pass through cell membrane through a large number of TFs to activate downstream functional genes. To date, a number of TF involved in metabolic pathway have been identified in different plants. Our review summarizes a network that is involved in the transcriptional regulation of PSM biosynthesis under environmental stresses (Figure 1). To date, a large number of positive TFs have been identified, but the number of negative TFs is still limited. Negative regulatory TFs are also the key factors in the establishment of dynamic balance of plant secondary metabolism. In the future research, cloning and identification of negative regulatory TFs has become an urgent research hotspot.

## Conclusion and Future Perspectives

Plants produce a large number of PSMs having diversified structures, and they play important physiological and ecological roles in stress tolerance. The biosynthesis of stress-related PSMs is controlled by environmental and genetic factors. Artificial regulation of PSM biosynthesis is helpful to enhance plant resistance to environmental stresses. We summarized potential genetic and environmental factors and their targets, particularly in MYB, bHLH, and WRKY families. For plant genetic improvement, overexpression of activating TFs or inhibition of expression of inhibitory TFs can increase the yield of PSM and enhance the resistance of plants to environmental stress. We further found that the downstream targets of these TFs are frequently enriched in the synthesis pathway of precursors, suggesting an effective role of precursors in enhancing of terminal products. Although most of PSM-related TFs have been identified in different plant species, including the model plant *Arabidopsis*, medicinal plant *C. roseus*, and woody plant poplar, these results also provide good guides for the regulation in other plants. This review summarizes the key enzymes and TFs involved in PSM biosynthetic pathways, providing valuable insights for screening targets and regulators in non-model plants.

## Author Contributions

RC was involved in the review writing. RC and ZC were involved in manuscript refinement. XZ and CS initiated the idea of the review and were involved in the manuscript writing. All authors contributed to the article and approved the submitted version.

## Conflict of Interest

The authors declare that the research was conducted in the absence of any commercial or financial relationships that could be construed as a potential conflict of interest.

## Publisher’s Note

All claims expressed in this article are solely those of the authors and do not necessarily represent those of their affiliated organizations, or those of the publisher, the editors and the reviewers. Any product that may be evaluated in this article, or claim that may be made by its manufacturer, is not guaranteed or endorsed by the publisher.
